# Trends in Surgical Treatment for cT4 Breast Cancer After Neoadjuvant Systemic Therapy: A Nationwide Study in The Netherlands

**DOI:** 10.1245/s10434-025-17585-2

**Published:** 2025-06-18

**Authors:** Britt A. M. Jansen, Johannes C. Kelder, Tim Borchert, Dominique J. P. van Uden, Femke van der Leij, Carolien Schröder, Annemiek Doeksen, Sabine Siesling, Marissa C. van Maaren, Emily L. Postma

**Affiliations:** 1 Department of Surgery, St. Antonius Hospital, Utrecht, The Netherlands; 2 Department of Statistical Analysis, St. Antonius Hospital, Nieuwegein, The Netherlands; 3 Department of Surgery, Canisius Wilhelmina Hospital, Nijmegen, The Netherlands; 4 Department of Radiation Oncology, University Medical Centre Utrecht, Cancer Centre, Utrecht, The Netherlands; 5 Department of Internal Medicine, University Medical Center Groningen, Groningen, The Netherlands; 6 Department of Health Technology and Services Research, Technical Medical Centre, University of Twente, Enschede, The Netherlands; 7 Department of Research and Development, Netherlands Comprehensive Cancer Organisation (IKNL), Utrecht, The Netherlands

**Keywords:** cT4 breast cancer, Neoadjuvant systemic therapy, Modified radical mastectomy, Surgical trends, Survival

## Abstract

**Background:**

Current guidelines recommend neoadjuvant systemic therapy (NST) followed by modified radical mastectomy (MRM) for stage T4 breast cancer. In this study, trends in MRM and de-escalated surgery of cT4a-c and cT4d breast cancer were evaluated and the impact of treatment on survival was assessed.

**Methods:**

Patients with cT4N_any_M0 breast cancer who received NST between 1989 to 2020 were selected from the Netherlands Cancer Registry. Rates of MRM and de-escalated breast/axillary surgery were analyzed for the periods 1989–1999, 2000–2009, and 2010–2020. Cox proportional hazard regression with inverse probability weighing was used to estimate for confounding-adjusted hazard ratios (HRs) for overall survival. Crude relative survival was calculated using excess mortality ratios from national life tables.

**Results:**

This study included 2,541 patients with cT4a-c and 1479 with cT4d breast cancer. The frequency of MRM decreased from 78% in 1989–1999 to 54% in 2010–2020 for cT4a-c and from 82% to 70% for cT4d patients. De-escalated surgery was associated with better overall survival than MRM, for both cT4a-c (HR 0.74, 95% confidence interval [CI] 0.63–0.87) and cT4d breast cancer (HR 0.78, 95% CI 0.63–0.96). Five-year crude relative survival for MRM versus de-escalated treatment was 66% (95% CI 0.64–0.69) versus 83% (95% CI 0.79–0.87) for cT4a-c, and 56% (95% CI 0.53–0.59) versus 70% (95% CI 0.64–0.76) for cT4d.

**Conclusions:**

Modified radical mastectomy rates decreased over time. De-escalated surgery was associated with improved 5-year overall survival compared with MRM. These findings suggest that de-escalated surgery is at least equivalent to MRM in terms of survival and may support consideration of less invasive surgical approaches.

**Supplementary Information:**

The online version contains supplementary material available at 10.1245/s10434-025-17585-2.

With a global incidence of 2.3 million cases diagnosed per year, breast cancer remains a significant health concern globally.^1^ Breast cancer includes a group of heterogeneous malignancies that can be categorized into different anatomical stages based on the tumor size and extent of spread.^2^ Accurate staging is important for determining appropriate treatment strategies. Although relatively rare, stage T4 breast cancer is the most locally advanced stage and is associated with the poorest prognosis.^3^ As defined by the American Joint Committee on Cancer (AJCC), T4 breast cancer is classified as a tumor of any size with direct extension to the chest wall and/or skin. T4 breast cancer is divided into noninflammatory locally advanced breast cancer (NI-LABC; T4a-T4c) and inflammatory breast cancer (IBC; T4d), together accounting for 5% of all new breast cancer diagnoses.^4,5^ In the case of clinical T4 (cT4) breast cancer, and cT4d specifically, international guidelines recommend trimodal therapy, which encompasses neoadjuvant systemic therapy (NST), modified radical mastectomy (MRM), and radiotherapy.^6–8^ Treatment strategies continuously evolve to optimize patient outcomes by balancing less invasive treatments, associated with improved quality of life, with the goal of achieving optimal oncological control.^9^

In the past years, NST has gained a crucial role in breast cancer treatment.^10^ One of the benefits of NST is the potential to decrease tumor size and stage of the disease in the breast and/or axilla.^11,12^ Pathological complete response (pCR) is the ultimate outcome of NST and is associated with better outcomes in terms of disease-free survival compared with patients who do not achieve pCR after NST.^13^ Besides being associated with more favorable prognoses, tumor size reduction, particularly a radiological complete response, and downstaging following NST allows surgeons to consider breast-conserving surgery (BCS) over total mastectomies.^14,15^ Additionally, for axillary surgery, less invasive options, such as the sentinel node (SN) procedure or marking the axilla with radioactive iodine seeds (MARI), have advanced to replace the traditional axillary lymph node dissection.

Multiple studies of current clinical practice have indeed shown a decrease in mastectomy and axillary lymph node dissection (ALND) rates after NST in early breast cancer.^16–18^ To date, there has been limited nationwide research investigating the impact of de-escalated surgical treatment in patients with cT4a-c and cT4d breast cancer. In this nationwide cohort study, we aimed to compare the baseline characteristics of patients with cT4a-c and cT4d breast cancer who underwent NST, delineate surgical trends in both groups, and assess their influence on sFurvival rates. We hypothesize that the use of mastectomy in combination with ALND (i.e., MRM) has decreased without affecting the survival rates of cT4.

## Methods

### Data Collection

For this retrospective cohort study, data were obtained from the Netherlands Cancer Registry (NCR), which is hosted by the Netherlands Comprehensive Cancer Organisation (IKNL). It contains population-based information on all newly diagnosed malignancies since 1989. Trained registration clerks register patient-, tumor-, and treatment-related characteristics directly from patient files. The primary source of notification is the Dutch Nationwide Pathology Databank (Palga).

### Patients

All women with cT4N_any_M0 breast cancer who received NST between 1989 to 2020 were selected from the NCR. Males, patients with irradical resections, cases with unspecified cT4 stage, or unknown surgical procedures were excluded from the analysis.

### Definitions

Tumor staging was based on TNM classification, of which criteria for T4 tumors did not change over the included years.^4^ cT4d diagnosis is based on a combination of physical examination, radiological imaging, and biopsy, with the final diagnosis being determined and documented in multidisciplinary consultation.

Histological breast cancer type and Bloom-Richardson differentiation grade were categorized according to the International Classification of Diseases for Oncology (ICD-O) standard. Hormone receptor status (estrogen-ER and progesterone-PR) and human epidermal growth factor receptor-2 (HER2) status were registered since 1995 and 2005, respectively. Pathological complete response (pCR) was defined as no residual invasive tumor in the breast (ypT0/is) and axilla (ypN0/is), with “y” indicating that the Tumor and Nodal classification were performed after NST. Vital status was verified using the municipal administration, including date of death, and was completed until February 2022.

Surgical procedures were categorized as follows:

1. Surgical treatment according to the Dutch guidelines includes MRM, which involves mastectomy in combination with ALND.
2. De-escalation in surgical treatment, including mastectomy in combination with SN or MARI procedure, mastectomy alone, BCS in combination with ALND, BCS in combination with SN or MARI, and BCS alone.

### Statistical Analysis

Baseline patient-, tumor-, and treatment-related characteristics were presented according to cT4 stage (i.e., cT4a-c and cT4d) and compared by using the Student’s *t*-test or Wilcoxon rank-sum test for continuous variables and the Pearson’s chi-square test for categorical variables.

The trends in surgical treatment, including breast and axilla, were illustrated using stacked bar graphs spanning from 1989 to 2020. To analyze potential differences in surgical treatment between time cohorts, patients were divided into three periods: 1989–1999, 2000–2009, and 2010–2020. Additionally, the trends in axillary treatment, including both surgical methods (with the most invasive technique presented) and radiotherapy modalities, were displayed for the period from 2011 to 2020, corresponding with the NCR registry’s initiation of recording specific sites of locoregional radiotherapy and SN procedures in 2011. To analyze potential differences in axillary treatment over time, patients were divided into the following periods: 2011–2014, 2015–2017, and 2018–2020. Since the NCR started recording MARI procedures in 2015, data for MARI procedure trends are considered reliable starting from that year. For patients with pCR, the trends of surgical treatment were shown from 1994–2020, because no patients had achieved pCR before 1994. A Pearson’s chi-square test was deployed to compare the treatment characteristics between the periods.

For survival analysis, patients were divided into two surgical treatment groups: MRM versus de-escalated surgical treatment. Cox proportional hazard regression was used to assess overall survival for both groups. Both the crude hazard ratio (HR), as well as the HR after stabilized inverse probability of treatment weighting (IPTW), were obtained. To balance clinicopathological characteristics between groups, age, histological type, differentiation grade, clinical nodal stage, (neo)adjuvant systemic therapy, hormone and HER2 receptor status, and pCR were used in a multivariable logistic regression model to calculate the probability of the type of treatment, resulting in propensity scores, as implemented in the “twang” package in R. By using the “twang” package, rates of missingness were balanced between treatment and control groups.^19,20^ Missing data were assumed to be missing at random.

The relative survival, i.e., the survival of the study cohort compared with the general population, which were matched on age, gender and calendar year, was estimated using the period approach and calculated with the Ederer II method, based on Dutch national life tables of Statistics Netherlands (www.cbs.nl).

*P*-values <0.05 were considered as statistically significant. Data analyses were performed using IBM SPSS Statistics v29 and R statistical software version 3.4.3.

## Results

### Patient Characteristics

**Fig. 1:**
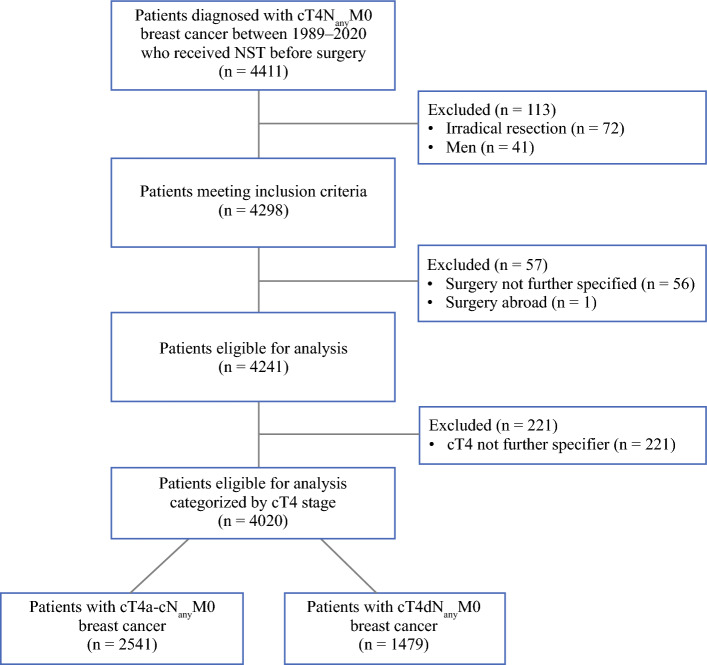
Cohort selection

From 1989 to 2020, a total of 4,411 patients were diagnosed with cT4N_any_M0 and were treated with NST before surgery, of which 4,020 remained after applying exclusion criteria (Fig. 1). Almost two-thirds of patients were diagnosed with cT4a-c (*n* = 2,541, 63.2%) and approximately one-third with cT4d (*n* = 1,479, 36.8%; Table 1). Patients with cT4d were significantly younger than patients with cT4a-c (54 vs. 60 years, *p* < 0.001). More cT4d patients were diagnosed with ductal carcinoma versus cT4a-c (76.9% vs. 73.8%, *p* < 0.001). Lymph node involvement (N1-3) was more common in patients with cT4d compared with cT4a-c (76.9% vs. 63.1%, *p* < 0.001). HER2-positive breast cancer was more common in cT4d than cT4a-c (26.9% vs. 16.8%, *p* < 001), and more patients with cT4d received neoadjuvant chemotherapy (93.4% vs. 78.4%, *p* < 0.001) and neoadjuvant targeted therapy (21.9% vs. 13%; *p* < 0.001), whereas most patients with cT4a-c received endocrine therapy (26% vs. 10.3%; *p* < 0.001). For both groups, most patients were treated with mastectomy and ALND (Table 1). Trimodal therapy was applied in 60.4% of patients with cT4a-c breast cancer and 70.3% of patients with cT4d breast cancer. Pathological complete response was more common in patients with cT4d (13.1%) compared with cT4a-c (6.5%, *p* < 0.001).

**Table 1:** Patient, tumor, and treatment characteristics of patients with T4 breast cancer (*n* = 4,020)

	cT4a-c	cT4d	*p*
*Patient and tumor characteristics*			
Patients, *N* (%)	2541 (63.2)	1479 (36.8)	
Age at diagnosis (yr), median (IQR)	60 (49–71)	54 (46–65)	<.001
Tumor type, *N* (%)			
Ductal	1874 (73.8)	1137 (76.9)	<.001
Lobular	258 (10.2)	95 (6.4)	
Other	409 (16.1)	247 (16.7)	
Grade, *N* (%)			
1	153 (6)	42 (2.8)	<.001
2	651 (25.6)	268 (18.1)	
3	641 (25.2)	473 (32)	
Missing	1096 (43.2)	969 (47.1)	
Clinical nodal stage (cN), *N* (%)			
N0	812 (32.0)	280 (18.9)	<.001
N1	1294 (50.9)	822 (55.6)	
N2	132 (5.2)	78 (5.3)	
N3	178 (7)	236 (16)	
NX	126 (4.9)	63 (4.3)	
Receptor status, *N* (%)			
HR+	1414 (55.6)	676 (45.7)	<.001
HR−	463 (18.2)	529 (35.8)	
Missing	664 (26.1)	274 (18.5)	
HER2+	426 (16.8)	398 (26.9)	
HER2−	1260 (49.6)	711 (48.1)	
Missing	855 (33.6)	370 (25)	
*Treatment characteristics*			
Neoadjuvant systemic therapy, *N* (%)			
Chemotherapy	1992 (78.4)	1382 (93.4)	<.001
Endocrine therapy	661 (26)	152 (10.3)	<.001
Targeted therapy	336 (13)	328 (21.9)	<.001
Adjuvant systemic therapy, *N* (%)			
Chemotherapy	183 (7.1)	131 (8.8)	<.001
Endocrine therapy	1766 (68.1)	763 (51)	<.001
Targeted therapy	330 (13)	326 (22)	<.001
Radiotherapy, *N* (%)			
Neoadjuvant	40 (1.5)	25 (1.7)	<.001
Adjuvant	2095 (82.4)	1285 (86.9)	
No radiotherapy	408 (16.1)	169 (11.4)	
Breast surgery, *N* (%)			
Mastectomy	2275 (89.5)	1421 (96.1)	<.001
Lumpectomy	267 (10.5)	58 (3.9)	<.001
Axillary surgery, *N* (%)			
ALND	1905 (75)	1206 (81.5)	<.001
SN / MARI	399 (15.7)	151 (10.2)	
No surgery	47 (1.8)	17 (1.1)	
Missing	190 (7.5)	105 (7.1)	
Trimodal therapy, *N* (%)	1530 (60.4)	1039 (70.3)	<.001
pCR (breast + lymph nodes), *N* (%)	166 (6.5)	194 (13.1)	<.001
Missing	415 (16.3)	265 (17.9)	
Breast cancer subtype, *N* (%)			
HER2+HR+	32 (12.9)	47 (22.8)	
HER2+HR−	61 (34.3)	71 (35)	
HER2−HR+	21 (2)	18 (4.1)	
HER2−HR−	36 (15.7)	47 (17.2)	

*ALND* axillary lymph node dissection; *SN* sentinal node; *MARI* marking the axilla with radioactive iodine seeds; *HR* hormone receptor status; *HER2* human epidermal growth factor receptor-2; *pCR* pathological complete response; *IQR* interquartile range

### Trends in Surgical Treatment in cT4a-c and cT4d

**Fig. 2:**
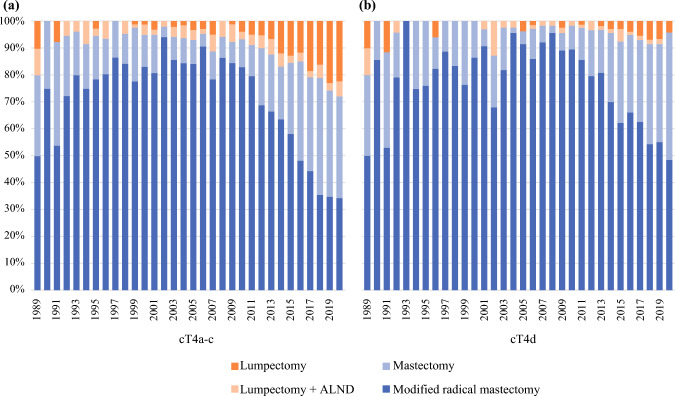
Trends of surgical treatment between 1989 and 2020 for **a** cT4a-c breast cancer (*n* = 2,541) and **b** cT4d breast cancer (*n* = 1,479)

Figures 2a and 2b show the trends of surgical treatment for the breast and axilla of cT4a-c and cT4d. For patients with cT4a-c, the MRM rates during the periods of 1989–1999, 2000–2009, and 2010–2020 were 78%, 85%, and 54%, respectively (*p* < 0.001). For cT4d these rates were 82.4%, 88.5%, and 69.5%, respectively (*p* < 0.001; Table 2).

**Table 2:** Surgical treatment characteristics of cT4a-c (*n* = 2541) and cT4d (*n* = 1479) in subgroups of incidence years: 1989–1999, 2000–2009, and 2010–2020

	cT4a-c				cT4d			
	1989–1999	2000–2009	2010–2020	*p*	1989–1999	2000–2009	2010–2020	*p*
*All patients*	*n* = 378	*n* = 999	*n* = 1,164		*n* = 159	*n* = 557	*n* = 763	
MRM	295 (78)	849 (85)	629 (54)	<.001	131 (82.4)	493 (88.5)	530 (69.5)	<.001
Patients with pCR	4 (1.4)	35 (4.1)	94 (14.9)		5 (3.8)	39 (7.9)	111 (20.1)	
De-escalation	83 (22)	150 (15)	535 (46)		28 (17.6)	64 (11.5)	233 (30.5)	
Patients with pCR	0 (0)	2 (1.3)	31 (5.8)		0 (0)	5 (7.8)	34 (14.6)	
*Patients with de-escalated breast surgery*	83 (22)	150 (15)	535 (46)		28 (17.6)	64 (11.5)	233 (30.5)	
Simple mastectomy	65 (78.3)	90 (60)	346 (64.7)		26 (92.9)	46 (71.9)	195 (82.2)	
Lumpectomy	18 (21.7)	60 (40)	189 (35.3)		2 (7.1)	18 (28.1)	38 (16.3)	
*All patients with pCR*	4 (1.1)	37 (3.7)	125 (10.4)		5 (3.1)	44 (7.8)	145 (18.7)	
Surgical treatment								
MRM	4 (100)	35 (94.6)	94 (75.2)	.021	5 (100)	39 (88.6)	111 (76.6)	.113
De-escalation	0 (0)	2 (5.4)	31 (24.8)		0 (0)	5 (11.4)	34 (23.4)	

*MRM* modified radical mastectomy; *pCR* pathological complete response Values are presented as *n* (%)

**Fig. 3:**
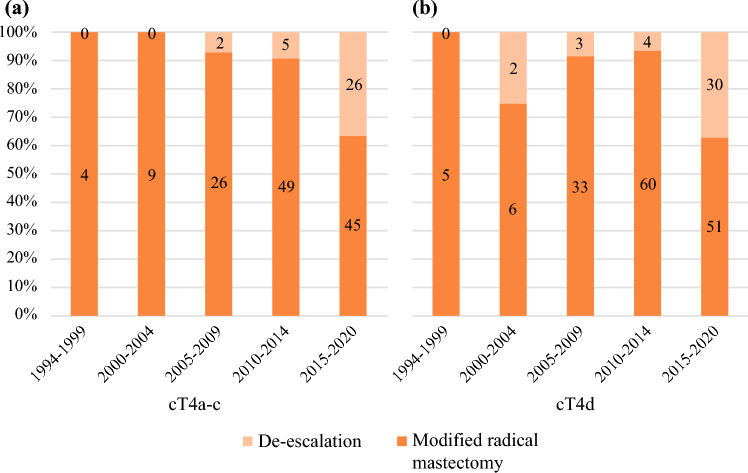
Trends of surgical treatment in patients with pathological complete response (pCR) between 1944 and 2020 in **a** cT4a-c breast cancer (*n* = 166) and **b** cT4d breast cancer (*n* = 194)

For patients with cT4a-c breast cancer with pCR (*n* = 166), a significant decrease in the use of MRM was observed over the periods (100%, 94.6%, and 75.2%, *p* = 0.021). For cT4d (*n* = 194), this trend was nonsignificant (100%, 88.6%, and 76.6%, *p* = 0.113; Figs. 3a and 3b; Table 2).

### Trends in Axillary Treatment in cT4a-c and cT4d

**Fig. 4:**
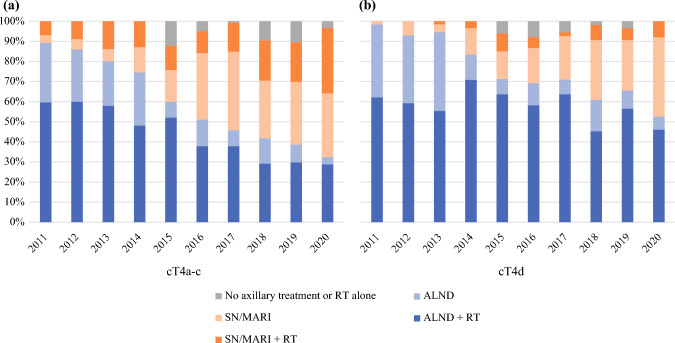
Trends of axillary treatment between 2011 and 2020 for **a** cT4a-c breast cancer (*n* = 1,060) and **b** cT4d breast cancer (*n* = 698).

For patients diagnosed with cT4a-c breast cancer since 2011 (*n* = 1,060), a significant decrease in the use of ALND was observed (74.8%, 52%, and 39.4%, *p* < 0.001), as well as for cT4d (*n* = 698; 82.4%, 69.5%, and 59.4%, *p* < 0.001; Table 3). A substantial increase in the use of SN and MARI procedures was observed (Figs. 4a and 4b). Additionally, the use of locoregional radiotherapy significantly increased from 57.7% to 68.5% (*p* < 0.001) for cT4a-c and from 66.4% to 77.2% (*p* < 0.001) in cT4d (Table 3).

**Table 3:** Axillary treatment characteristics of cT4a-c (*n* = 1,060) and cT4d (*n* = 698) in subgroups of incidence years: 2011–2014, 2015–2017, and 2018–2020

	cT4a-c				cT4d			
	2011–2014	2015–2017	2018–2020	*p*	2011–2014	2015–2017	2018–2020	*p*
*All patients*	*n* = 416	*n* = 342	*n* = 302		*n* = 301	*n* = 200	*n* = 197	
Surgical treatment								
ALND, n (%)	311 (74.8)	178 (52.0)	119 (39.4)	<0.001	248 (82.4)	139 (69.5)	117 (59.4)	<0.001
De-escalation, n (%)	105 (25.2)	164 (48.0)	183 (60.6)		53 (17.6)	61 (30.5)	80 (40.6)	
Radiotherapy treatment								
Locoregional	240 (57.7)	257 (75.1)	207 (68.5)	<0.001	200 (66.4)	168 (84.0)	152 (77.2)	<0.001
Local	95 (22.8)	50 (14.6)	60 (19.9)		55 (18.3)	21 (10.5)	20 (10.2)	
No RT	54 (13.0)	33 (9.6)	29 (9.6)		33 (11.0)	9 (4.5)	13 (6.6)	
Missing	27 (6.5)	2 (0.6)	6 (2)		13 (4.3)	2 (1)	12 (6)	

*ALND* axillary lymph node dissection; *RT* radiotherapy

### Survival Outcomes

After IPTW, the multivariable Cox regression analyses showed that de-escalated treatment was significantly associated with better overall survival, for both cT4a-c (hazard ratio [HR] 0.74, 95% confidence interval [CI] 0.63–0.87) and cT4d breast cancer (HR 0.78, 95% CI 0.63–0.96; Table 4).

**Table 4:** Estimated hazard ratios in patients with cT4a-c and cT4d breast cancer treated with modified radical mastectomy versus de-escalated surgical treatment

		*n* (%)	Univariable analysis			IPTW analysis^a^		
			HR	95% CI	*p*	HR	95% CI	*p*
cT4a-c	Modified radical mastectomy	1773 (69.8)	Ref			Ref		
	De-escalated surgical treatment	768 (30.2)	0.7	0.62–0.8	<0.001	0.74	0.63–0.87	<0.001
cT4d	Modified radical mastectomy	1154 (78)	Ref			Ref		
	De-escalated surgical treatment	325 (22)	0.7	0.58–0.84	<0.001	0.78	0.63–0.96	0.02

*CI* confidence interval; *IPTW* inverse probability treatment weighting; *HR* hazard ratio ^a^Corrected for age, morphology, differentiation grade, HR/HER2 status, cN stadium, (neo)adjuvant chemotherapy, endocrine therapy, and targeted therapy, radiotherapy, and pCR

The 5-year crude relative survival for cT4a-c breast cancer improved from 49.7% to 59.4% to 79.6% in the time periods 1989–1999, 2000–2009, and 2010–2020, respectively. For cT4d breast cancer, the rates increased from 35.1% to 47.8% to 67.8%, respectively (Table S1).

The crude 5-year relative survival rates of patients treated with MRM compared with patients with de-escalated treatment were 66% (95% CI 0.64–0.69) versus 83% (95% CI 0.79–0.87) for cT4a-c breast cancer, and 56% (95% CI 0.53–0.59) versus 70% (95% CI 0.64–0.76) for cT4d breast cancer (Table 5).

**Table 5:** Five-year relative survival outcomes of patients with cT4a-c and cT4d breast cancer treated with modified radical mastectomy versus de-escalated surgical treatment

		Crude relative survival^a^	
		5-year	95% CI
cT4a-c	Modified radical mastectomy	0.66	0.64–0.69
	De-escalated surgical treatment	0.83	0.79–0.87
cT4d	Modified radical mastectomy	0.56	0.53–0.59
	De-escalated surgical treatment	0.7	0.64–0.76

CI confidence interval

^a ^Relative survival at 5 years using the period approach and calculated with the Ederer II method

## Discussion

Although trimodal therapy has been recognized as the standard treatment for cT4 breast cancer, notable surgical trends have emerged. In this study, data from 4,091 patients with cT4 breast cancer treated with NST were analyzed to reflect the surgical trends and their impact on survival rates. Within this Dutch population, a significant decrease in the proportion of patients undergoing MRM was observed. Simultaneously, this de-escalation of surgical procedures did not adversely influence breast cancer survival outcomes.

Improved systemic strategies, including the shift from adjuvant to neoadjuvant therapies and the use of breast tumor-specific treatment modalities, resulted in tumor size reduction and downstaging and increased rates of pCR in both the affected breast and axilla.^21^ This is supported by our data, which show a clear increase in pCR rates over time (1.1% to 10.4% for cT4a-c and 3.1% to 18.7% for cT4d). The availability of contemporary, effective systemic therapy offers the opportunity for breast and axillary conservation in patients with T4 breast cancer.^22–24^ However, in our study, even during the most recent decade (2010–2022), more than 75% of patients with pCR still underwent MRM.

Although there is some debate regarding the oncological safety of performing BCS compared with mastectomy, several studies have demonstrated that BCS is a safe option for cT4a-c.^24,25^ A recent review, in which the role of BCS in cT4d was explored, concluded that no data was supporting the use of BCS in cT4d.^23^ A study by Muzaffar et al., in which 7043 patients with cT4d were analyzed found that patients treated with mastectomy had better 5-year and median survival rates compared with those who underwent BCS.^26^ However, patients in the BCS group were more likely to have characteristics frequently associated with poorer outcomes, such as older age and non-White ethnicity. Additionally, these studies all contain data from patients treated before 2013, whereas our cohort includes data from more recent years and thus better reflects current clinical practice with improved diagnostic and therapeutic options.

While SN is widely accepted for axillary staging, its accuracy has not been proven across all breast cancer patient groups. In cases of T4 tumors, ALND remains indicated, even though SN is already commonly performed.^27^ Preoperative chemotherapy can compromise the accuracy of SN procedure owing to altered lymphatic drainage patterns, leading to increased false-negative rates.^28,29^ Therefore, ALND remains necessary to adequately stage the axilla in postneoadjuvant patients with cT4 breast cancer. In a large registry study, 1917 patients with cT3/T4a-c, N0 tumors were analyzed to compare survival rates, showing a survival benefit associated with ALND.^30^ Also for cT4d, ALND is still recommended regardless of the response to NAC, as there is no evidence supporting the use of SN. However, with the increasing efficacy of NST and radiotherapy, the importance of axillary staging may be diminished, allowing for the possibility of de-escalating axillary surgery, similar to lower tumor stages.^18,31^

Despite our effort to adjust for potential confounders in the overall survival analysis, we must consider the possibility of unmeasured confounding (e.g., comorbidities and patients’ preference). The observation of higher survival rates in patients undergoing de-escalated surgical treatment suggests that this group has more favorable patient and tumor characteristics, although only partially adjusted for. Given that pCR is a strong prognostic factor for improved survival, it would be expected to act as a major confounder. However, in addition to adjusting for pCR in our multivariate analysis, patients in the de-escalation groups did not have higher pCR rates compared with those in MRM groups. Nevertheless, our findings align with the growing body of observational data, which show improved oncological outcomes with BCS compared with mastectomy in patients with locally advanced breast cancer treated with NST.^32,33^

As per guidelines, postoperative radiotherapy is usually indicated for cT4 breast cancer.^34^ Notably, 14.5% of all patients did not receive radiotherapy. Patients within this subset tended to be older, were more frequently diagnosed with triple-negative breast cancer, received less neoadjuvant chemotherapy, and were more likely to receive neoadjuvant endocrine therapy; one-third were part of the de-escalated treatment group (data not shown). We suspect that the omission of radiotherapy does not necessarily indicate a de-escalation strategy but is rather an approach focused on local disease control through surgery and/or systemic therapy. Our results show that this approach was more common in the past than in recent years, indicating a historical tendency towards interventions aiming not only for symptom relief but also prevention of future complications. This shift over time reflects evolving perspectives and practices within this field. Also, 1.6% of patients received neoadjuvant radiotherapy, which is not in accordance with current guidelines, but this was primarily in earlier years (median 1,989; interquartile range 1,995–2,003).^35^

This research is subjected to some limitations. First, challenges arise in analyzing cT4d, because it lacks a specific biomarker and remains a clinical diagnosis with wide variability in presentation among individuals. Consequently, patients might be unintentionally misclassified as cT4d because of differences in subjective categorization.^36^ Similarly, many cases of cT4d may have been missed because clinical symptoms are not always recognized. For this reason, in 2022 Jagsi et al. proposed a quantitative diagnostic scoring system to identify cT4d.^36^ The baseline characteristics of cT4d patients in this cohort align with baseline data from other cT4d studies, indicating accurate classification of the evaluated patients. Tumor-characteristics of cT4d patients in our cohort show similarities to those reported in other studies, for example, the higher proportion of patients with HER2-receptor positivity (26.9% vs. 16.8%), grade 3 (31.7% vs. 25.2%), and nodal involvement (81.2% vs. 67.9%) within the cT4d group compared with cT4a-c.^6,37^ Therefore, we presume that minimal misclassification has occurred in our cohort. Second, several well-known predictors of survival (i.e., HER2, estrogen, and progesterone receptor status) are missing in earlier years as these tumor characteristics were not assessed routinely yet. Additionally, data on radiological complete response were not available, which is important because these patients are likely to have undergone de-escalation surgery. To account for this, we chose to adjust for pCR, because it is probable that the response was already visible on preoperative imaging in these patients. Another important missing data point is the reason for de-escalation, including the patients’ perspective. Breast cancer-specific survival could not be determined, because we did not have information on the cause of death. Therefore, we conducted a relative survival analysis, which is an approximation of the disease-specific survival, although we did not correct for confounding in these analyses. Additionally, data on (locoregional) recurrence were not available in our dataset. This represents a limitation, particularly in the light of the 2018 EBCTCG meta-analysis, which reports increased locoregional recurrence following NST.^38^ Lastly, information regarding comorbidities, patient preferences, and the contribution of shared-decision making was not available, which may influence the type of surgical treatment received.

Although patients with a radiological complete response following NST may be suitable candidates for future de-escalation studies, conducting clinical trials on surgical de-escalation in this context may be challenging and potentially unfeasible owing to the large sample sizes required and the need for long follow-up periods to assess long-term outcomes.^10^ However, further high-quality data are needed to evaluate the impact of de-escalation strategies on recurrence and survival and to support informed shared decision-making for patients with cT4 tumors.

## Conclusions

This study demonstrates a shift towards less aggressive surgical interventions in patients diagnosed with cT4 breast cancer. Both cT4a-c and cT4d cases have shown an increasing adoption of BCS and omission of ALND, without negatively impacting oncological outcomes. While preliminary studies suggest potential for surgical de-escalation, current evidence is insufficient to conclude that treatment can safely be deescalated, particularly in T4d breast cancer. It is important to emphasize that de-escalation in T4d breast cancer is likely to be safe only in patients who achieve pCR. Future research should focus on more accurately identifying which patients are suitable candidates for these approaches and how such strategies impact quality of life.

## Supplementary Information

Below is the link to the electronic supplementary material.

Supplementary file1 (DOCX 15 KB)
