# Supplementary material for: Trends in Surgical Treatment for cT4 Breast Cancer After Neoadjuvant Systemic Therapy: A Nationwide Study in The Netherlands
Source: Ann Surg Oncol. 2025 Jun 18;32(12):8668–77. doi: 10.1245/s10434-025-17585-2 (PMC12534241; doi:10.1245/s10434-025-17585-2)
Supplement: Supplementary file 1 — Supplementary file1 (DOCX 15 KB) [file 10434_2025_17585_MOESM1_ESM.docx]

**Supplementary material**

| **Table S1**. Five-year relative survival outcomes of patients with cT4a-c and cT4d breast cancer in subgroups of incidence years: 1989-1999, 2000-2009, and 2010-2020. | | | |
| --- | --- | --- | --- |
|  |  | **Relative survival ^a^** | |
|  |  | **5 year (%)** | **95% CI** |
| **cT4a-c** | 1989-1999 | 49.7 | 41.3 – 58.1 |
|  | 2000-2009 | 59.4 | 55.3 – 63.5 |
|  | 2020-2020 | 79.6 | 76.4 – 82.8 |
| **cT4d** | 1989-1999 | 35.1 | 23.6 – 46.6 |
|  | 2000-2009 | 47.8 | 42.2 – 53.4 |
|  | 2020-2020 | 67.8 | 63.7 – 71.9 |
| *Abbreviations*: CI = confidence interval. | | | |
| ^a^ Relative survival following Ederer II method at 5 years | | | |
